# Silicon modifies C:N:P stoichiometry and improves the physiological efficiency and dry matter mass production of sorghum grown under nutritional sufficiency

**DOI:** 10.1038/s41598-022-20662-1

**Published:** 2022-09-27

**Authors:** Jonilson Santos de Carvalho, Joaquim José Frazão, Renato de Mello Prado, Jonas Pereira de Souza Júnior, Milton Garcia Costa

**Affiliations:** 1 State University of Southwest Bahia (UESB), Estrada do Bem Querer, Km 04, Vitória da Conquista, 45031-300 Brazil; 2 School of Agricultural and Veterinarian Sciences, São Paulo State University (Unesp), Via de Acesso Prof. Paulo Donato Castellane s/n, Jaboticabal, 14884-900 Brazil

**Keywords:** Plant development, Plant physiology

## Abstract

Silicon (Si) may be involved in the modification of C:N:P stoichiometry and in physiological processes, increasing sorghum growth and grain production. The objective was to evaluate the effect of Si supply on C:N:P:Si stoichiometry, physiological response, growth, and grain production of sorghum. The experiment was carried out in pots with four concentrations of Si: 0; 1.2; 2.4; and 3.6 mmol L^−1^ in a completely randomized design, with six replicates. Physiological attributes and dark green color index were measured and grain and biomass production were determined. Posteriorly, the plant material was ground to determine silicon (Si), carbon (C), nitrogen (N), and phosphorus (P) contents in order to analyze C:N:P:Si stoichiometry. C:Si and C:N ratios decreased at all Si concentrations applied (1.2, 2.4, and 3.6 mmol L^−1^) and in all plant parts studied, being lower at 3.6 mmol L^−1^. The lowest C:P ratios of leaves and roots were observed at 3.6 mmol L^−1^ Si and the lowest C:P ratio of stems was observed at 1.2 mmol L^−1^ Si. Si concentrations were not significant for the N:P ratio of leaves. The highest N:P ratio of stems was observed at 3.6 mmol L^−1^, while the lowest N:P ratio of roots was observed at 2.4 and 3.6 mmol L^−1^. Regardless of photosynthetic parameters, the application of 1.2 mmol L^−1^ Si enhanced photosynthetic rate. The application of 2.4 and 3.6 mmol L^−1^ enhanced stomatal conductance and dark green color index. The mass of 1000 grains was not influenced by Si applications, while Si applications at all concentrations studied (1.2, 2.4, and 3.6 mmol L^−1^) enhanced shoot and total dry matter, not affecting root dry matter and grain production. In conclusion, Si supply modifies C:N:P:Si stoichiometry and increases physiologic parameters, growth, development, and grain production in sorghum.

## Introduction

The use of silicon (Si) in agriculture has direct economic benefits for increasing yield and having a lower relative cost than conventional fertilizers. Furthermore, very diluted Si solutions (< 3 mmol L^−1^) are used in fertigation due to its high efficiency, greatly reducing the amount of silica required in a given area and providing economic benefits^1–3^. Si, an important beneficial element for plants^4–6^, is absorbed as monosilicic acid, being present in plant tissues. Its main functions can be classified as structural, morphological, and physiological^6–9^, with a more significant effect on plants that accumulate the element, such as grain sorghum (*Sorghum bicolor* L. Moench)^8–11^.

Sorghum plants have specific transporters for Si absorption (such as LSi_1_ and LSi_2_) which are highly efficient in the root absorption of Si^11^, thus including sorghum in the group of Si-accumulating plants^10^. In addition, studies indicated that silicate fertilization mitigates nutritional stresses such as ammonium toxicity^12^, boron deficiency^13,14^, and manganese deficiency^15^. Furthermore, Si can regulate the levels of endogenous plant hormones under stress conditions, such as abscisic acid and gibberellins^16^^.^

The structural role played by Si is related to the incorporation of the beneficial element into cell walls, such as opals, which are also called phytoliths (SiO_2_ nH_2_O)^8,10^. After Si absorption, some of the organic compounds can be replaced by the incorporation of Si in plant tissues, decreasing carbon (C) content and consequently influencing the stoichiometry of C:N:P:Si in different tissues (leaves, stems, and roots), as verified in *Phragmites australis*^17^, sugarcane^18–20^ and forages ^21^.

The substitution of C for structural Si in the cell wall stands out as one of the main advantages of silicate fertilization for plants, as it represents energy savings of up to 80%^22^. The value of one mole of ATP per mole of SiO_2_ deposited is admitted as the cost of silicification^18^, while five moles of ATP and two moles of NADPH per mole of C assimilated are adopted as the minimum requirement for other growth processes^23^. This energy saving may optimize carbon use efficiency by inducing a stoichiometric homeostasis involving carbon (C), nitrogen (N), and phosphorus (P), which are structural nutrients, thus favoring carbon metabolism and the growth and grain production of sorghum, although proof is needed^24^.

The increase in phytolith concentration influences cell resistance and the maintenance and integrity of plant vessels^11,25^. In addition, Si also contributes to improve leaf architecture by increasing absorption, sunlight use efficiency, and pigment production^9^.

Carbon uptake can vary by light period, photosynthetic activity and cell cycle, and high photosynthetic activity occurs after cell division while the rate of photosynthesis for cells was low prior to division^26^. On the other hand, silicon uptake and deposition appear to be associated with the formation of new siliceous valves just prior to cell division, and thus mainly seem to be confirmed to the periods between cytokinesis and daughter cell separation^27^. The variation in the Si/C, observed in some early studies^18,20,28,29^, showed the different regulation mechanisms involved in Si and C metabolism and the uncoupling of Si and C incorporation^30^. In addition Si/C ratio can vary due to abiotic stress^31^, such as drought^32,33^, light intensity^34,35^, nutritional disorder^35,36^, salt stress^37^ and temperature^31^.

Among the main physiological benefits of supplying Si to plants, the increase in chlorophyll content^38,39^, stomatal conductance, and photosynthetic activity are highlighted^9,40^. Once absorbed, Si is deposited in the cells of the leaf epidermis and in guard cells of the stomata, forming a Cuticle-Si double layer^11^, which may affect gas exchanges. In the literature, information on the effect of Si on gas exchanges is contradictory.

Given these biological benefits of Si in plants, it is known that it is a mitigating agent of different stresses in several species, including sorghum^9,25,41–43^. In plants under nutritional sufficiency, it is possible that Si may also benefit their physiological attributes. However, energy savings may be occurring in plants before the application of Si by decreasing the C content in plant tissues, as aforementioned for some species. These plants may be optimizing the use of carbon by inducing a stoichiometric homeostasis involving carbon, nitrogen, and phosphorus, which are structural nutrients, thus favoring carbon metabolism and the growth of sorghum plants, although proof is needed.

Given the above, it is assumed that Si can be a vital element in optimizing the productivity of sorghum, which is a crop with nutritional importance for being a source of protein and energy, besides being a gluten-free and low-cost. Sorghum is mainly cultivated in hot and dry regions around the world, in large-scale commercial operations and small farms^44^. In this context, the hypothesis that the supply of Si modifies the C:N:P:Si stoichiometry, favoring the physiological responses and improving the growth and grain production of sorghum (*Sorghumbicolor* L. Moench) cultivated under nutritional sufficiency arises.

Once these hypotheses are accepted, it will be possible to better understand the biological role played by Si in the elementary stoichiometric homeostasis of the sorghum culture and support the expansion of the use of Si in the culture, ensuring an increase in the production of sustainable biomass, as there are many regions with soils that present low Si contents available, which is an element that presents no risks to the environment. Therefore, this research was carried out aiming to evaluate the effect of Si supply via nutrient solution (roots) on C:N:P:Si stoichiometry and to verify its effect on grain production and physiological responses in grain sorghum plants.

## Results

### Si, C, N, and P concentration and accumulation in sorghum plant parts

**Figure 1:**
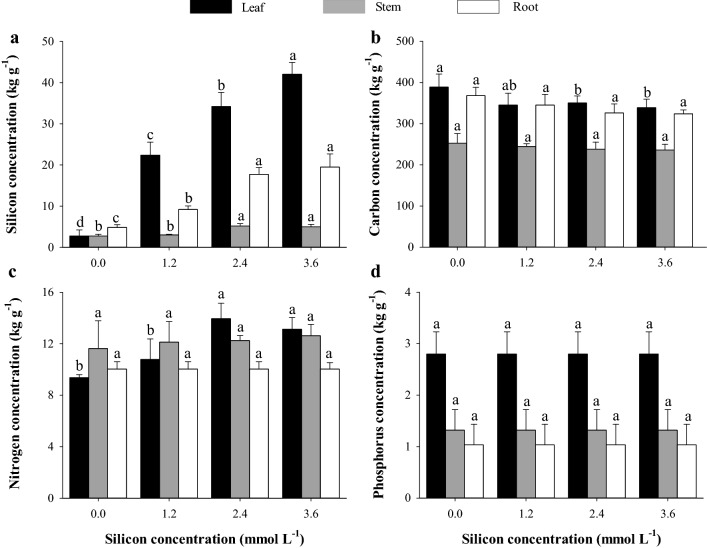
Concentration of silicon (Si) (**a**), carbon (C) (**b**), nitrogen (N) (**c**), and phosphorus (P) (**d**) in different parts of grain sorghum plants under different Si concentrations. Error bars over the dots indicate the standard error of the mean, *, **, and ns—significant at 1%, at 5%, and not significant by the F test, respectively.

The lowest Si concentration in leaves, stem and roots was observed in treatment control (0.0 mmol L^−1^). The application of 1.2 mmol L^−1^ Si increase leaves and root Si concentration but did not differ from the treatment control for Si concentration in stem (Fig. 1a). The highest Si concentration applied (3.6 mmol L^−1^) resulted in an increase of Si in the leaves of sorghum plans, while did not differing from the Si concentration of 2.4 mmol L^−1^ in the stems and roots of sorghum plants (Fig. 1a).

The application of 2.4 and 3.6 mmol L^−1^ Si decreased the concentration of C (Fig. 1b) in sorghum leaves, while not influencing the concentration of these elements in the stems and roots. On the other hand, the application of 2.4 and 3.6 mmol L^−1^ Si increased the concentration of N (Fig. 1c) in sorghum leaves. The concentration of P in the leaves, stems, and roots of sorghum, in turn, was not influenced by the application of Si at the concentrations studied (Fig. 1d).

**Figure 2:**
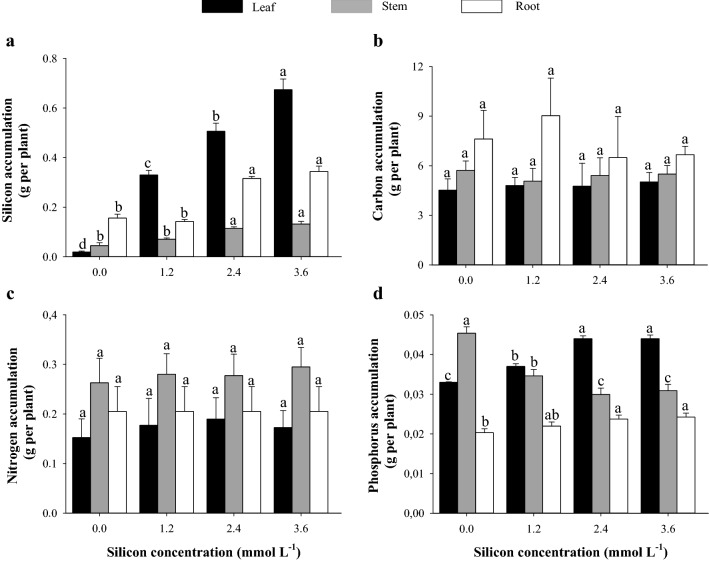
Accumulation of silicon (Si) (**a**), carbon (C) (**b**), nitrogen (N) (**c**), and phosphorus (P) (**d**) in different parts of grain sorghum plants under different Si concentrations. Error bars over the dots indicate the standard error of the mean. *, **, and ns—significant at 1%, at 5%, and not significant by the F test, respectively.

The highest Si accumulation was observed in leaves of plants that received 3.6 mmol L^−1^ Si, and in stem and roots of plants that received 2.4, and 3.6 mmol L^−1^ Si (Fig. 2a). The accumulation of C (Fig. 2b) and N (Fig. 2c) in all plant parts did not differ between the different concentrations of Si applied to sorghum plants. The highest concentrations of Si (2.4 and 3.6 mmol L^−1^ Si) applied increased P accumulation in leaves and roots and reduced P accumulation in the stem of sorghum (Fig. 2d).

### Stoichiometric relationships

**Figure 3:**
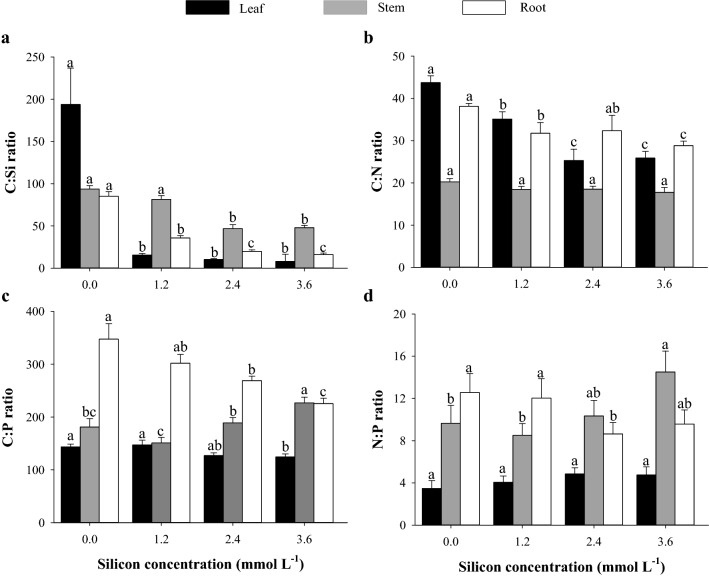
C:Si (**a**), C:N (**b**), C:P (**c**), and N:P (**d**) ratios in different parts of grain sorghum plants under different silicon (Si) concentrations. Error bars over the dots indicate the standard error of the mean. *, **, and ns—significant at 1%, at 5%, and not significant by the F test, respectively.

The highest C:Si ratio (Fig. 3a) was observed in the leaves and roots of sorghum plants in the control treatment (0.0 mmol L^−1^ Si) and in the stem of sorghum plants in the treatments at Si concentrations of 0.0 and 1.2 mmol L^−1^. The C:N ratio (Fig. 3b) decreased as the Si concentration increased, reaching the lowest value in leaves at the concentrations of 2.4 and 3.6 mmol L^−1^ Si and in the stem at the concentration of 3.6 mmol L^−1^ Si, with no effect on the roots. The C:P ratio (Fig. 3c) in the leaves and roots of sorghum plants presented the lowest value at the concentration of 3.6 mmol L^−1^ Si, while this Si concentration resulted in the highest C:P ratio in the stems. The N:P ratio (Fig. 3d) in leaves was not influenced by Si application. However, the N:P ratio in the stem was higher in plants that received 3.6 mmol L^−1^ Si; while being higher in the roots of plants that received the lowest Si concentrations, i.e., 0.0 and 1.2 mmol L^−1^.

### Physiological responses and production

**Figure 4:**
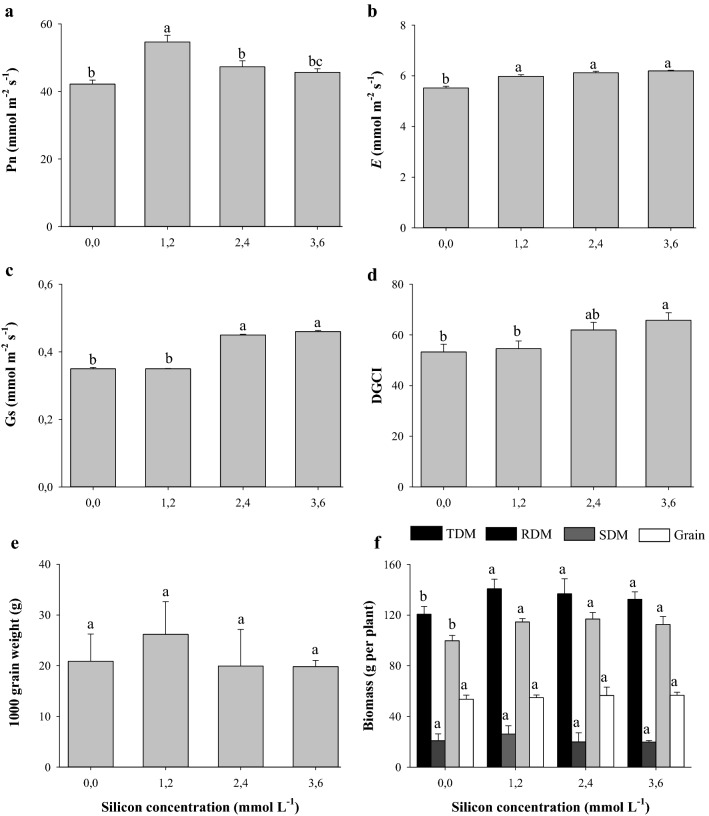
Photosynthetic rate (*A*) (**a**), transpiration (*E*) (**b**), stomatal conductance (*gs*) (**c**), dark green color index (DGCI) (**d**), thousand grain weight (**e**), and biomass production (grains; root: RDM; shoot: SDM, total: TDM) (**f**) of grain sorghum plants under different silicon (Si) concentrations. Error bars over the dots indicate the standard error of the mean. *, **, and ns—significant at 1%, at 5%, and not significant by the F test, respectively.

Si application at a concentration of 1.2 mmolL^−1^ resulted in the highest photosynthetic rate (Pn) (Fig. 4a). Leaf transpiration reached higher values in the treatments in which plants received Si, without difference between Si concentrations and the control treatment (Fig. 4b). The highest value for stomatal conductance was observed at the concentrations of 2.4 and 3.6 mmol L^−1^ Si (Fig. 4c); while the highest green color index was observed at the concentration of 3.6 mmol L^−1^ Si (Fig. 4d). The mass of 1000 grains (Fig. 4e) and grain and root biomass production (Fig. 4f) were not influenced by Si application of Si. However, Si application at all concentrations studied increased shoot and total biomass production in relation to the control treatment (Fig. 4f).

**Figure 5:**
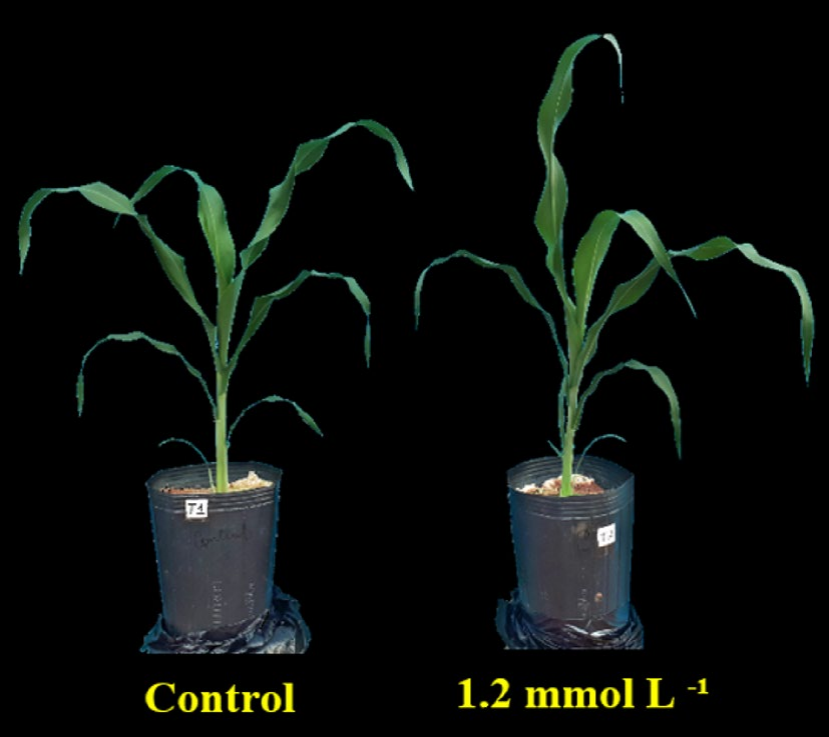
Grain sorghum plants cultivated with nutrient solution without Si (control) and with Si (1.2 mmol L^−1^) in pots filled with sand at 75 DAS.

Regarding leaf architecture, it is possible to observe that plants that received Si (1.2 mmol L^−1^) had leaves with a lower degree of inclination in relation to the stem, that is, more erect leaves, a fact that did not occur with leaves in the control treatment (without Si) (Fig. 5). This effect of Si could contribute to a more efficient capture of light, consequently favoring photosynthesis and biomass production.

Silicon benefited the stoichiometry and physiology of sorghum, despite showing no effect for the mass of 1000 grains (Fig. 4e), as it increased shoot dry mass and total biomass production (Fig. 4f). Through polynomial regression, it is possible to estimate the best concentration of Si for root dry matter production (y = − 0.94x^2^ + 2.63x + 21.75, R^2^ = 0.92**), shoot dry matter production (y = − 3.3x^2^ + 15.3x + 100.14, R^2^ = 0.99**), and grain production (y = − 3.2x^2^ + 14.3x + 46.4; R^2^ = 0.99**). In this polynomial study, root (RDM) and shoot (SDM) biomass production increased until 1.40 mmol L^−1^ and 2.31 mmol L^−1^ Si, reaching 23.5 g and 117.9 g. Total dry matter production (TDM) increased until 117.9 g, reached at 2.14 mmol L^−1^ Si. Grain production also increased with quadratic adjustment as a function of Si application, with the maximum point being 62.4 g, reached at 2.23 mmol L^−1^ Si.

### Multivariate analysis

#### Hierarchical clustering analysis

**Figure 6:**
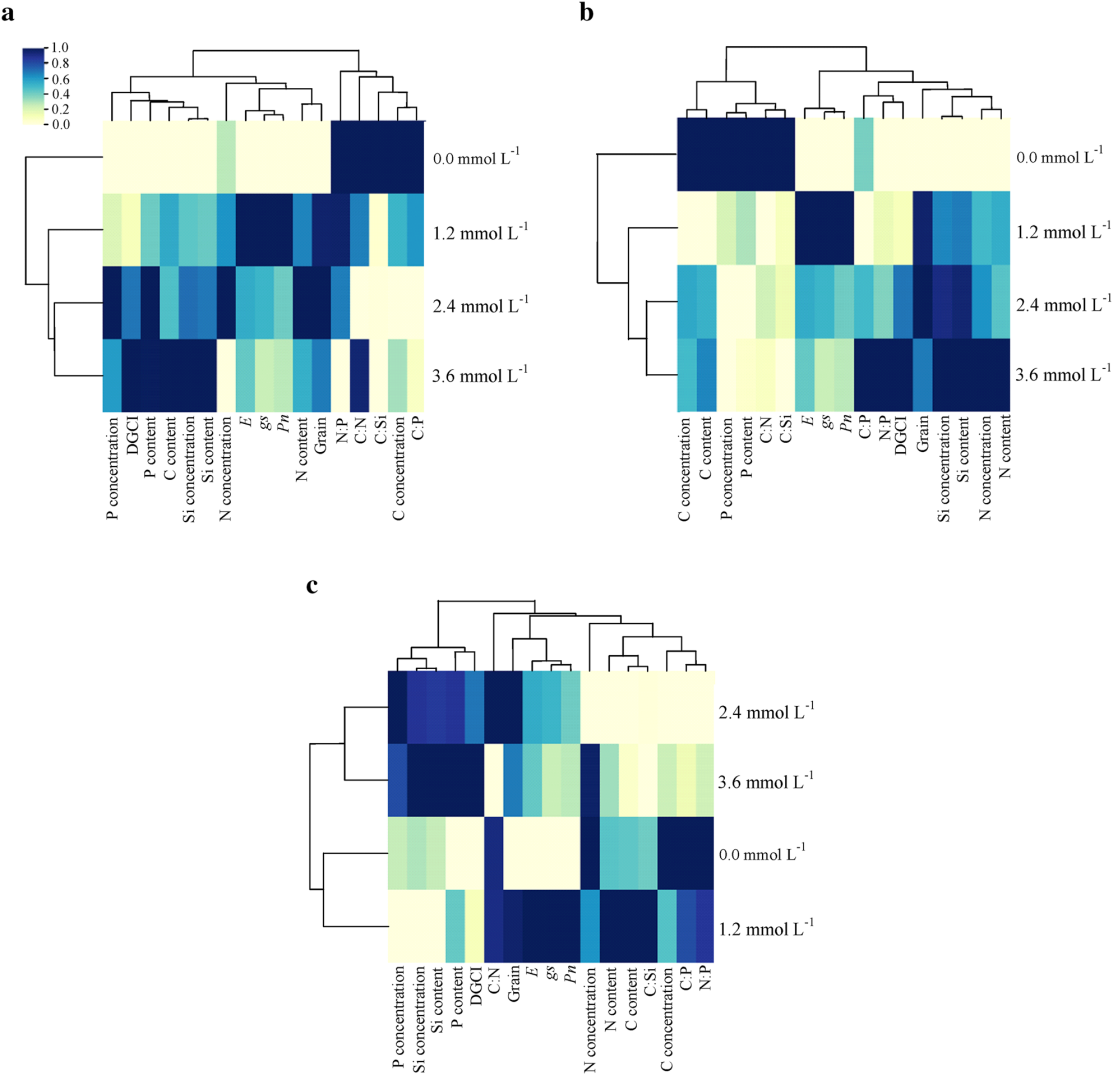
Hierarchically clustered heat map of the variables of concentration, accumulation, and stoichiometric ratios of C:N:P:Si in the leaf (**a**), in the stem (**b**), and in the root (**c**) associated with the physiological variables and the biomass of grain sorghum plants under different concentrations of silicon (Si). 0.0 to 1.0 indicates similarity between variables, where 1.0 is the greatest similarity and 0.0 the greatest dissimilarity.

The hierarchical clustering analysis of leaves resulted in the formation of two groups was verified; the first group was formed by the isolated subgroup of N:P and the subgroup of C:N, C:Si, C concentration, and C:P (Fig. 6a). The second group was divided into the following two subgroups: concentrations and accumulations of P and Si, accumulation of C and DGCI; and concentration and accumulation of N, *E, gs,* Pn, and grain biomass. In addition, high dissimilarity was observed for stoichiometric ratios (N:P, C:N, C:Si, C:P) and the C concentration at.4 mmol L^−1^ Si, which is the concentration that showed greater similarity with grain biomass. Physiological responses (*E*, *gs*, and Pn) increased as a function of Si application, expressing greater similarity at the concentration of 1.2 mmol L^−1^, while the accumulation and concentration of N showed greater similarities at the concentration of 2.4 mmol L^−1^. Furthermore, the variables DGCI, Si concentration, and accumulation of P, C and Si were favored by greater similarity with the concentration of 3.6 mmol L^−1^ Si (Fig. 6a). In the stem, the first group consisted of concentration and accumulation of C and P and the stoichiometric ratios of C:N and C:Si, while the second group consisted of the subgroup of *E, gs*, and Pn and the subgroup of the C:P and N:P stoichiometric ratios, concentrations, and accumulation of Si and N, DGCI, and grain biomass (Fig. 6b). The responses of concentrations and accumulations of C and P and the C:N and C:Si stoichiometric ratios showed similarity with the concentration of 0.0 mmol L^−1^ Si, while the concentrations and accumulations of N and Si and C:P and N:P stoichiometric ratios were favored at the highest Si concentration of Si (3.6 mmol L^−1^).

The clustering analysis of roots showed the formation of the two following groups for the treatments: the first group consisted of the concentrations of 2.4 and 3.6 mmol L^−1^ Si and the second group consisted of the concentrations of 0.0 and 1.2 mmol L^−1^ Si (Fig. 6c). Regarding the independent variables in the root, there was the formation of the two following groups: the first group consisted of the concentrations and accumulations of P and Si and the DGCI and the second group consisted of the subgroup with C:N ratio, *E, gs,* Pn, and grain biomass and the second subgroup, with the concentrations and accumulations of C and N and the stoichiometric ratios of C:Si, C:P, and N:P. It was also found that the C concentration and the stoichiometric ratios of C:P and N:P in the root expressed greater similarity with the 0.0 mmol L^−1^ Si concentration, while the concentration and accumulation of N were more favored by the concentration of 1.2 mmol L^−1^ Si (Fig. 6c). However, P concentration and C:N ratio expressed greater similarity with the 2.4 mmol L^−1^ Si concentration, while Si and P accumulations were more favored by the Si concentration equal to 3.6 mmol L^−1^ Si (Fig. 6c).

#### Principal component analysis (PCA)

**Figure 7:**
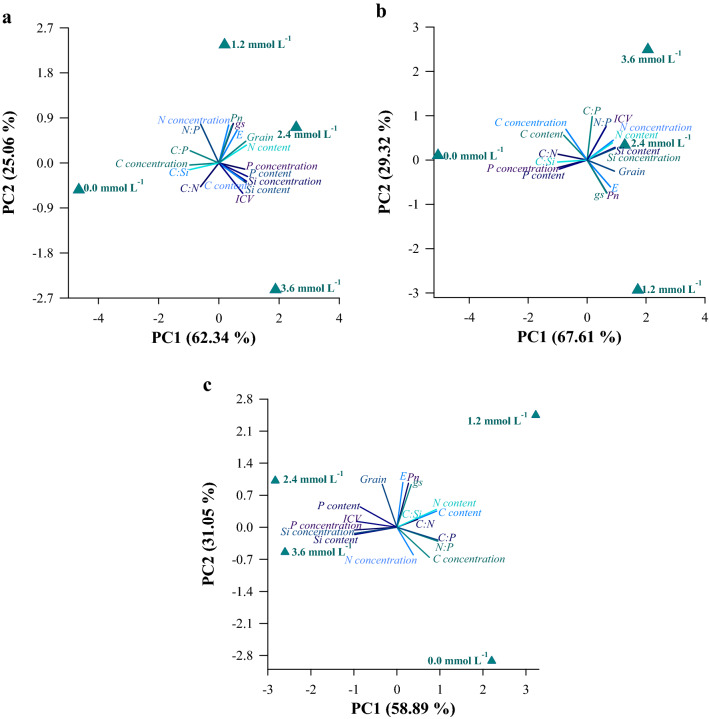
Principal component analysis of the C:N:P:Si concentration, accumulation, and stoichiometric ratios in the leaf (**a**), stem (**b**), and root (**c**) variables associated with physiological variables and grain yield of grain sorghum plants under different silicon (Si) concentrations.

Principal components 1 and 2 explained 89%, 97%, and 90% of the results for leaves (Fig. 7a), stems (Fig. 7b) and roots (Fig. 7c), respectively. For PCA in leaves, Si, P, and C concentrations; C, N, P, and Si accumulations; C:P and C:Si ratios; dark green color index (DGCI); and grain ratios contributed to explain the variance in PC1, while N concentration; N:P ratio; *gs*; and Pn contributed to explain the variance in PC2. The C:N and *E* ratio contributed with mean values ​​to explain the variance in PC1 and PC2 (Fig. 7a; Table 1). In the stem, C, N, P, and K concentrations; N, P, and Si accumulations; C:N and C:Si; *E* and grain ratios contributed to explain the variance in PC1, while C:P ratio; DGCI; *gs*; and Pn contributed to explain the variance in PC2. C concentration, and N:P ratio contributed to explain the variance in PC1 and PC2 (Fig. 7b; Table 1). For the root, it was found that Si and P concentrations; C, N, P, and Si accumulation; C:P, N:P, and C:Si ratios; and DGCI contributed to explain the variance in PC1, while N concentration, *E, gs*, Pn, and grain ratios contributed to PC2.C concentration and C:N ratio contributed with mean values to explain the variance in PC1 and PC2 (Fig. 7c; Table 1).

**Table 1:** Factor loadings extracted from independent variables in leaf (a), stem (b) and root (c) of grain sorghum plants under different Si concentrations.

Variable	Leaf		Stem		Root	
	PC1	PC2	PC1	PC2	PC1	PC2
Si concentration	0.9181	− 0.3666	0.9479	0.2852	− 0.9827	− 0.1601
N concentration	0.3287	0.7586	0.8862	0.4472	0.3802	− 0.6008
P concentration	0.8616	− 0.1246	− 0.9707	− 0.1667	− 0.9845	− 0.0598
C concentration	− 0.9637	− 0.0425	− 0.7970	0.5599	0.7534	− 0.6564
Si accumulation	0.9044	− 0.3969	0.9510	0.2631	− 0.9838	− 0.1347
N accumulation	0.9146	0.3564	0.8589	0.3839	0.8553	0.3267
P accumulation	0.9534	− 0.2717	− 0.9469	− 0.2062	− 0.8532	0.4459
C accumulation	0.8017	− 0.3465	− 0.7171	0.6915	0.9253	0.3529
C:N ratio	− 0.6044	− 0.4754	− 0.9865	0.1301	0.4807	0.1705
C:P ratio	− 0.9510	0.2431	0.1727	0.9800	0.9520	− 0.3061
N:P ratio	− 0.6015	0.7742	0.6287	0.7354	0.9550	− 0.2787
C:Si ratio	− 0.9620	− 0.1349	− 0.9988	− 0.0470	0.9175	0.3916
DGCI	0.7925	− 0.6048	0.6464	0.7554	− 0.9326	0.1294
E	0.6027	0.6507	0.7928	− 0.6073	0.1406	0.9776
gs	0.4829	0.7876	0.6705	− 0.7414	0.2680	0.9634
Pn	0.4322	0.7439	0.6596	− 0.7428	0.3339	0.9337
Grains	0.8950	0.4340	0.9237	− 0.2507	− 0.3424	0.9395

The PCA of leaves indicated a relationship between physiological variables (*E, gs*, and Pn), concentration and accumulation of N, and grain biomass, with concentrations of 1.2 and 2.4 mmol L^−1^ Si, while C concentration and stoichiometric ratios (N:P, C:P, C:Si, and C:N) showed an association in the 0.0 mmol L^−1^ Si concentration and the variable responses of P and Si concentrations, accumulations of C, P, and Si and DGCI were related with the concentration of 3.6 mmol L^−1^ Si.

For the stem, PCA indicated that the concentration and accumulation of Si were related with the concentrations of 1.2 and 2.4 mmol L^−1^ Si (Fig. 7b). It was also verified that the concentration and accumulation of N in the stem were more strongly related with the concentrations of 2.4 and 3.6 mmol L^−1^ Si, while the stoichiometric ratios of C:P and N:P were associated with a higher Si concentration (3.6 mmol L^−1^). The PCA of roots indicated that concentrations and accumulations of P and Si were associated with the concentrations of 2.4 and 3.6 mmol L^−1^ Si (Fig. 7c). Furthermore, it was found that the stoichiometric ratios of C:Si and C:N in the root were strongly related to the concentration of 1.2 mmol L^−1^ Si.

## Discussion

The efficiency of sorghum in the root absorption of Si, as indicated by the increase in the content (Fig. 2a) and accumulation (Fig. 3a) of Si as the Si concentrations applied increased has been reported in several papers, mainly for plants under stress conditions^44,45^. Sorghum plants have specific transporters for Si absorption (such as LSi_1_ and LSi_2_) which are highly efficient in the root absorption of Si^46^, thus including sorghum in the group of Si-accumulating plants^10^. In addition, Si is absorbed and transported following the transpiration flow, accumulating mainly in organs with high transpiration rate, such as leaves, which are the main sites of Si accumulation in plants^47^.

The increase in the concentration and accumulation of Si in plants is related to changes in the concentrations and accumulation of other nutrients. Our study showed that the application of Si reduces the concentration of C (Fig. 1a). These results indicate the replacement of C by Si in organic compounds in the leaf cell wall, which is important due to the lower energy cost for Si incorporation compared to carbon compounds, resulting in metabolic energy savings of 10 to 20-fold compared to the incorporation of C^48^.

The effect of increasing Si concentrations and N concentrations in plants is controversial in the literature. While there is evidence that Si reduces N uptake by competition for carrier sites present in roots^45^, other papers indicate that Si increases N uptake^49^. The interaction between Si and N seems to depend on the species and the plant organ studied, since for sorghum plants there is a reduction in the concentration of N only in leaves (Fig. 1c).

Regarding P, although there are reports in the literature stating that Si increases P absorption, as Si increases the expression of genes related to inorganic P transporters in sorghum^50^, our results show no effect of Si application in the P concentration of leaves, roots, and stems of sorghum (Fig. 1d).

The greater absorption of Si in sorghum plants induced the modification of C:N:P stoichiometric homeostasis, decreasing C:Si (leaves, stems, and roots), C:N (leaves, stems, and roots), and C:P (leaves and roots) ratios and increasing the N:P ratio (leaves and roots) (Fig. 2).

It is known that Si can modifies C:N:P:Si in plants under stress conditions. Under drought stress it is known that Si increase root water and nutrient uptake; invokes plant defense responses and regulates leaf water loss^51^. In heat stress situation, Si increases the activity of antioxidant enzymes, preventing damage by reactive oxygen species accumulation and preventing lipid peroxidation; reduces abscisic acid and salicylic acid levels; improves water uptake and, consequently, leaf water status; and improve photosynthetic rates^52^. Silicon also improves pigments production, photosynthetic rates and morphological characteristics of roots, enhancing development, yield and quality of soybean under shading stress^53^.

Our results reveal that the benefits of Si in modifying C:N:P stoichiometric homeostasis are not restricted to stressed plants, as reported in sorghum cultivation under saline stress^39^. The alteration of C:N:P stoichiometric homeostasis is caused partially by the biological function of Si, which partially replaces C in organic compounds in cell walls for lower energy costs^41^. Additionally, it is hypothesized that Si increases water absorption and transpiration^54,55^, also stimulating H^+^ATPase membrane activity and inducing nutrient uptake^49^. In this study, it is evidenced that Si absorption decreased C:Si (leaves, stems, and roots), C:N (leaves, stems, and roots), and C:P (leaves and roots) ratios, while increasing the N:P ratio (leaves and roots) (Fig. 2).

In a recent study, Frazão et al.^18^ and Souza Junior et al.^20^ observed a decrease in C concentration in different sugarcane tissues as the Si supply increased, with a subsequent reduction in C:Si, C:N, and C:P ratios, as also observed in this study (Fig. 3a,b,c).

Although it is indicated some problems with using whole tissue analyses Si and C trade-offs^56^, the substitution of C for structural Si in the cell wall seems to be an advantage of silicate fertilization for plants, as it can represents energy savings of up to 80%^22^. The value of one mole of ATP per mole of SiO_2_ deposited is admitted as the cost of silicification^18^, while five moles of ATP and two moles of NADPH per mole of C assimilated are adopted as the minimum requirement for other growth processes^23^. This energy saving may optimize carbon use efficiency by inducing a stoichiometric homeostasis involving carbon (C), nitrogen (N), and phosphorus (P), which are structural nutrients, thus favoring carbon metabolism and the growth and grain production of sorghum, although proof is needed^24^.

It is indicated that Si cannot engage in as many chemical bonds with as C, and it forms mostly silicates and SiO_2_ polymers^57^, and, in addition, some studies indicates that Si per se does not promote plant growth, function or metabolic activity in plants under no stress conditions^58^. In addition, Hu et al.^59^ verified that Si can increase carbon content in canola plants. Nonetheless, some recent studies show that Si has physiological benefits and can modifies the stoichiometry C:N:P in plants under no stress conditions, such as sugarcane^18,20^, in quinoa^29^ and grassland^28^. In addition, Costa et al.^32^, in a recent study on the impact of Si on C, N and P stoichiometric homeostasis in sugarcane, verified that Si can change in the stoichiometric ratio C:N:P even in plants under ideal cultivation condition. The authors report that the application of Si, in plants with no stress, increases P and N uptake and the use efficiency of C, N and P. The information about the effect of Si in the C content and in the C, N and P stoichiometric homeostasis in sorghum remains unknown.

Unlike other stoichiometric ratios, the N:P ratio increased as the Si supply increased (Fig. 3d), resulting in a higher N concentration in the plant (Fig. 1c) compared to the P concentration (Fig. 1d). This was also observed in other species as a function of Si supply, such as in quinoa plants^50^.

The C:P and N:P ratios in the stem were associated with increasing Si concentrations (Figs. 6b and 7b). This may have occurred as there is a translocation of metabolic energy gains to the leaves, decreasing the P concentration in sorghum stems and consequently increasing the C:P and N:P ratios of sorghum stems.

Si is also known to increase the biosynthesis of photosynthetic pigments such as chlorophylls and carotenoids^54,60^, which explains the benefit of Si at the concentration of 3.6 mmol L^−1^ compared to control plants by increasing the dark green color index (DGCI) by 23%, which is considered as an indirect measure of chlorophyll content (Fig. 4d). The greater production of pigments, in turn, resulted in a trend towards a higher photosynthetic rate (Pn) (Fig. 4a), which contributed to obtaining a greater production of grain biomass (Fig. 4f). This increase in plant physiological activity promoted by Si was contributed by the increased excitation of the photosystem II reaction centers^54,61^, favoring photosynthetic efficiency. In addition, Si deposition in the plant tissue, known as opal phytoliths, adds strength and rigidity to cell walls^11^, consequently modifying plant architecture by making them more erect (Fig. 5), which may have contributed to the trend towards increasing Pn (Fig. 4a) and biomass production (grains, roots, and shoots) (Fig. 4f) due to the greater capacity of light interception by the leaf surface^47^.

Through multivariate analysis, the Pn, grain biomass, and the concentration and accumulation of foliar N showed high similarity (Fig. 6a), as N participates in the synthesis and composition of chlorophyll and is essential in the synthesis of phosphoenolpyruvate carboxylase (PEPC-enzymes) and ribulose-1,5-bisphosphate carboxylase oxygenase (RuBisCO)^62^. The enzyme RuBisCO plays the role of carboxylase when it catalyzes the initial carbon fixation step in the Calvin cycle, also playing the role of oxygenase in the photorespiration process. Meanwhile, phosphoenolpyruvate carboxylase (PEPcase) binds to HCO_3_^−^ to form oxaloacetate and inorganic phosphate (Pi)^62^. On the other hand, Si can also cause an increase in the concentration and accumulation of N from changes in primary metabolism, promoting the translocation of amino acids from the source of the synthesis to other plant tissues^49^. In the literature, information on the effect of Si on gas exchange is contradictory and may be related to the species, environmental conditions^63^, and concentrations used. Changes in morphophysiological characteristics as a response to changes in cell rigidity by the action of Si, consequently providing changes in plant growth, have already been verified in other species such as rice^61^ and sugarcane^64^. On the other hand, excess Si induces a thick layer, due to the saturation of this element, an increase in leaf phytoliths, and a reduction in stomatal opening, hindering the diffusive process of gas exchange^65,66^. Thus, the reductions in *Pn* values from the concentration of 1.9 mmol L^−1^ Si (Fig. 4a), associated with decreases in the accumulation of C in the plant (Fig. 2b), consequently decreased total biomass production (TDM, Fig. 4f). Additionally, the lowest mean TDM from this Si concentration onwards, associated with the stabilization of nitrogen (N) and phosphorus (P) concentration (Fig. 1c,d), also contributed to the absence of effects of Si on N and P accumulations in the plant (Fig. 2c,d).

The relevance of Si in elemental stoichiometry involving C in sorghum plants is better understood with a multivariate study. The reduction in the C concentration in the plant (Fig. 1b) also influenced the clustering of variables, and C:Si, C:N, and C:P stoichiometric ratios showed greater dissimilarity as the Si concentration increased, with these variables being in distant groups in leaves (Fig. 6a), stems (Fig. 6b), and roots (Fig. 6c). The greatest distance between C:Si, C:N, and C:P stoichiometric ratios as a function of the Si concentration, obtained by the cluster analysis of leaf variables (Fig. 6a), was observed at the concentration of 2.4 mmol L^−1^ Si. On the other hand, in this same Si concentration, it was also observed that the increase in the production of grain biomass is related to a decrease in the C:Si, C:N, and C:P stoichiometric ratios. The beneficial effects of Si on stoichiometric ratios, photosynthetic processes, and grain biomass production is reinforced by the hierarchical grouping of treatments in leaves and stems, indicating the formation of two dissimilar groups (without and with Si fertilization) as a result of the biological action of Si in sorghum. The results reinforce the importance of Si even in plants without the occurrence of stress, showing that it should be included among the elements that have a great influence on plant life.

In the present research, we unveiled that the optimal stoichiometric homeostasis in grain sorghum plants at the Si concentration of 2.2 mmol L^−1^, which resulted in the optimal production of grain biomass, is associated with stoichiometric ratios that varied according to the analyzed organ, as follows: C:Si (leaf: 38; stem: 63; and root: 32), C:N (leaf: 31; stem: 19; and root: 32), C:P (leaf: 133; stem: 168; and root: 273) and N:P (leaf: 4; stem: 11; and root: 11).

Therefore, our research indicated that Si promoted the homeostasis of structural nutrients that are known to be vital for the physiological process, such as N, as part of chlorophyll and enzymes, and P, as a constituent of nucleotides, phospholipids, and other compounds^50^. This directly favored the efficient use of C, which is evidenced by the increase in photosynthesis and the consequent, increase in the growth and production of biomass in the sorghum crop induced by its high capacity for Si absorption, thus confirming the hypothesis of this research.

The direct implication of this research is that the indication and expansion of the use of Si in the sorghum crop, even when under nutritional sufficiency, should favor the production of grain biomass or the sustainable productivity of this species without causing risks to the environment, especially in soils with low Si contents, which predominantly occurs in regions where this crop is grown.

Nevertheless, the main mechanisms involved in the responses of grain sorghum under different Si concentrations still need to be further explored, and studies at the proteomic level are suggested to understand the role played by Si in the expression of genes involved in the absorption and efficiency of use of C, N, and P, that is, in the metabolism of these elements that are so important to the physiological processes of plants.

The study showed that grain sorghum is responsive to Si up to a concentration of 2.2 mmol L^−1^ by increasing the N:P stoichiometric ratio and decreasing the C:Si, C:N, and C:P ratios, regardless of the plant part, at the same time favoring physiological aspects of sorghum. Thus, it is unveiled that the benefit of Si in sorghum plants under nutritional sufficiency occurs due to the induction of a new C:N:P stoichiometric homeostasis, consequently increasing the metabolic efficiency and optimizing the efficiency of the use of C, increasing photosynthesis and biomass and grain production.

It should be noted that the results of this study demonstrate the potential response of sorghum plants to the application of Si. However, this response depends on the Si concentration used. Among the Si concentrations analyzed in this paper, 2.2 mmol L^−1^ stood out. This paper provides information on the proper usage of Si in sorghum for future research involving this element.

## Material and methods

### Growth conditions and experimental design

**Figure 8:**
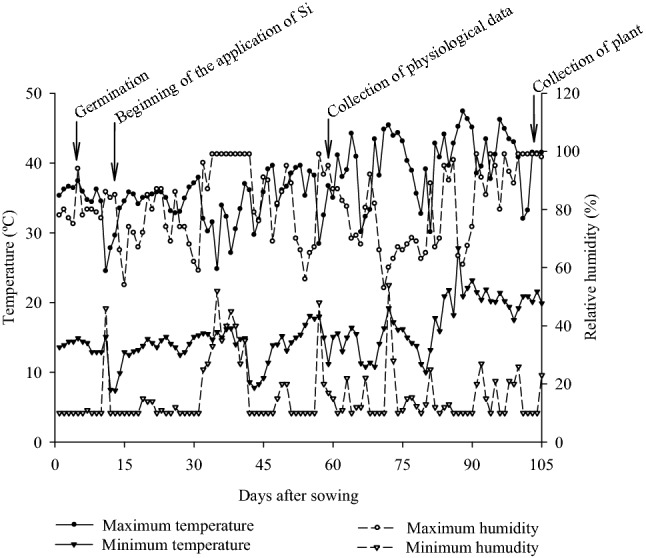
Maximum and minimum temperature (°C) and maximum and minimum relative humidity (%) recorded during the development of the experiment.

An experiment was carried out in a greenhouse with grain sorghum using a soilless cultivation system. During the development of the experiment, temperature and relative humidity were recorded using a thermo-hygrometer, obtaining means of 35.9 ± 11.4 °C; 19.3 ± 8.5 °C; 76 ± 23%; and 32 ± 22% for maximum temperature and minimum temperature and maximum relative humidity and minimum relative humidity, respectively (Fig. 8).

Treatments consisted of the following four Si concentrations: 0; 1.2; 2.4; and 3.6 mmol L^−1^, arranged in a completely randomized design, with six replicates. The Si source used was potassium silicate (SiK), Diatom^®^ (128 g L^−1^ Si; 126.5 g L^−1^ K_2_O and pH 12). Si was added to the nutrient solution^46^, the Fe source was modified to Fe-EDDHMA, and the pH value of the solution was adjusted between 5.5 and 6.5 with the addition of the HCl solution (1 mol L^−1^), being immediately supplied to the plants. The potassium content between treatments was balanced by adding KCl.

The experimental unit consisted of a polypropylene pot with capacity of 7 dm^3^ (upper diameter: 16 cm; lower diameter: 11 cm; height: 33 cm) filled with 6 dm^3^ of sand previously washed with running water, HCl solution (1%), and deionized water. Six seeds were distributed per experimental unit and thinning was carried out 21 days after emergence, maintaining one plant per pot.

The plants were irrigated with deionized water after sowing up to five days after emergence (DAE). Then, of a nutrient solution^67^ at 10, 20, 30, 40, 50, 60, and 70% of the concentration (ionic strength) were applied at 5, 12, 19, 26, 33, 40, and 47 DAE. From 47 DAE, we used 70% of the concentration until the end of the experiment. The nutrient solution was applied to maintain 70% of the substrate water holding capacity. The nutrient solution was applied until the nutrient solution started to drip from the bottom of the pot, indicating the need to interrupt its supply to prevent its loss.

The substrate was drained once a week to eliminate excess salts. During substrate drainage, 700 mL of deionized water was added to each pot, inducing the drainage of 400 mL of nutrient solution, which was discarded. After two hours, a new nutrient solution was added to the plants.

### Leaf gas exchange and dark green color index

At 60 days after sowing (DAS), in the morning (between 9 and 11 h), physiological attributes were evaluated in the middle third of the first fully expanded leaf.

The physiological evaluations consisted of non-destructive analyses in which the net photosynthetic rate (*A*), transpiration (*E*), and stomatal conductance (*gs*) were measured under saturating light using a portable system to measure CO_2_ and CO_2_ exchanges (LcPro-SD, ADC BioScientific Ltd., Hoddesdon, UK) at a constant luminous intensity of 1200 µmol m^−2^ s^−1^ emitted by a blue/red LED light source. Leaf temperature was maintained between 32.63 ± 0.77 °C and measurements were taken with the natural concentrations of CO_2_ in the air (between 400 ppm).

In addition, the dark green color index was measured with using the OptiSciences^®^ CCM-200 chlorophyll content meter, whose measurement unit is CCI (chlorophyll content index), which presents the value that is proportional to the amount of chlorophyll in the sample.

### Evaluations of biomass production and shoot dry matter

After the physiological maturation of the grains, panicles were harvested to determine yield with biomass being adjusted to the water content of 13%, and a mass of one thousand grains.

On the same occasion, the plants were harvested two centimeters above the substrate surface and divided into leaf, stem, and root. The collected material was washed in running water, neutral detergent (Extran^®^ 1%), acidic solution (HCl 1%), and distilled and deionized water, placed in paper bags, and submitted to a forced air ventilation oven at 65 ± 5 °C until the stability of the dry mass was obtained. Afterwards, the samples were weighed on a precision balance.

### Chemical analyses and stoichiometry

After weighing, 110 days after sowing, the material was ground in a Wiley mill and subjected to chemical analysis. Nitrogen (N) and phosphorus (P) contents were analyzed according to Bataglia et al.^68^. Carbon analysis (C) was performed using the Walkley–Black method with external heat, according to Tedesco et al.^69^. The silicon content (Si) was determined according to the methodology described by Korndörfer et al.^70^. The accumulation of C, N, P, and Si was calculated from the product of dry mass by its respective concentration in each plant tissue evaluated.

### Statistical analysis

Data were subjected to analysis of variance (*p* < 0.05) and, when significant, to Tukey’s test (*p* < 0.05). Univariate analyzes were performed using Sisvar49. Multivariate analysis of hierarchical clustering and principal components was performed, using the similarity coefficient and simple-linkage clustering as group connection algorithms for hierarchical cluster analysis and the data correlation matrix for principal component analysis. For the processing of multivariate analysis, the Python programming language (version 3.9.7; Python Software Foundation) was used.

## Data Availability

The datasets used and/or analysed during the current study available from the corresponding author on reasonable request.
